# 10,10′-Methyl­enebis[2,3-dihydro-1*H*-benzo[*e*]pyrrolo[1,2-*a*][1,4]diazepine-5,11(10*H*,11a*H*)-dione] dihydrate

**DOI:** 10.1107/S1600536809040501

**Published:** 2009-10-10

**Authors:** Hanane Benzeid, Nathalie Saffon, Bernard Garrigues, El Mokhtar Essassi, Seik Weng Ng

**Affiliations:** a Laboratoire de Chimie Organique Hétérocyclique, Pôle de Compétences Pharmacochimie, Université Mohammed V-Agdal, BP 1014 Avenue Ibn Batout, Rabat, Morocco; b Service Commun Rayons X, Université Paul Sabatier, Bâtiment 2R1, 118 route de Narbonne, 31062 Toulouse, France; c Hétérochimie Fondamentale et Appliquée, Université Paul Sabatier, UMR 5069, 118 Route de Narbonne, 31062 Toulouse, France; d Department of Chemistry, University of Malaya, 50603 Kuala Lumpur, Malaysia

## Abstract

The organic mol­ecule and uncoordinated water mol­ecule in the crystal of the title compound, C_25_H_24_N_4_O_4_·2H_2_O, both lie on special positions of twofold symmetry. A twofold rotation axis passes through the methyl­ene C atom connecting the two dihydro­benzopyrrolodiazepindionyl parts. The seven-membered C_5_N_2_ ring adopts a boat conformation.

## Related literature

Pyrrolo[2,1-*c*][1,4]benzodiazepines are a group of potent chemicals produced by *Streptomyces *species. For their anti-cancer activity, see: Bose *et al.* (1992 ▶); Cargill *et al.* (1974 ▶); Gregson *et al.* (2004 ▶).
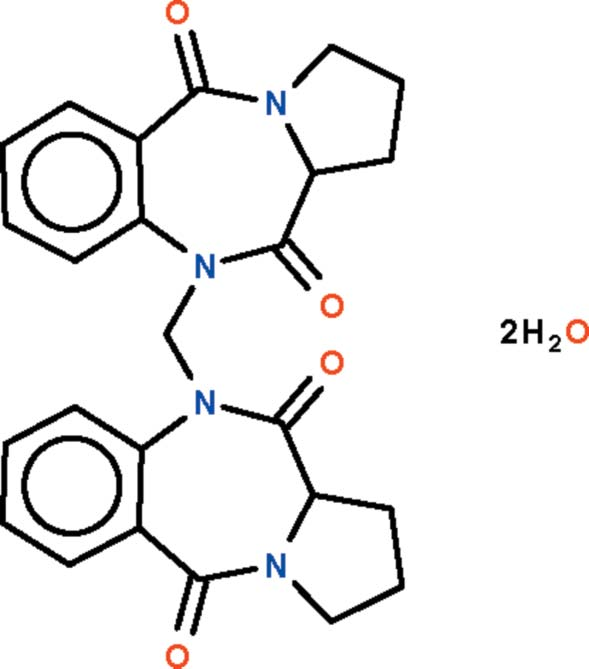


## Experimental

### 

#### Crystal data

C_25_H_24_N_4_O_4_·2H_2_O
*M* *_r_* = 480.51
Trigonal, 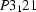

*a* = 11.9901 (2) Å
*c* = 13.6054 (2) Å
*V* = 1693.90 (4) Å^3^
*Z* = 3
Mo *K*α radiation
μ = 0.10 mm^−1^
*T* = 193 K
0.20 × 0.20 × 0.10 mm

#### Data collection

Bruker APEXII diffractometer
Absorption correction: none
23041 measured reflections
1572 independent reflections
1472 reflections with *I* > 2σ(*I*)
*R*_int_ = 0.043

#### Refinement

*R*[*F* ^2^ > 2σ(*F* ^2^)] = 0.043
*wR*(*F* ^2^) = 0.126
*S* = 1.18
1572 reflections
159 parameters
H-atom parameters constrained
Δρ_max_ = 0.46 e Å^−3^
Δρ_min_ = −0.48 e Å^−3^

### 

Data collection: *APEX2* (Bruker, 2005 ▶); cell refinement: *SAINT* (Bruker, 2005 ▶); data reduction: *SAINT*; program(s) used to solve structure: *SHELXS97* (Sheldrick, 2008 ▶); program(s) used to refine structure: *SHELXL97* (Sheldrick, 2008 ▶); molecular graphics: *X-SEED* (Barbour, 2001 ▶); software used to prepare material for publication: *publCIF* (Westrip, 2009 ▶).

## Supplementary Material

Crystal structure: contains datablocks global, I. DOI: 10.1107/S1600536809040501/tk2545sup1.cif

Structure factors: contains datablocks I. DOI: 10.1107/S1600536809040501/tk2545Isup2.hkl

Additional supplementary materials:  crystallographic information; 3D view; checkCIF report

## Figures and Tables

**Fig. 1.**
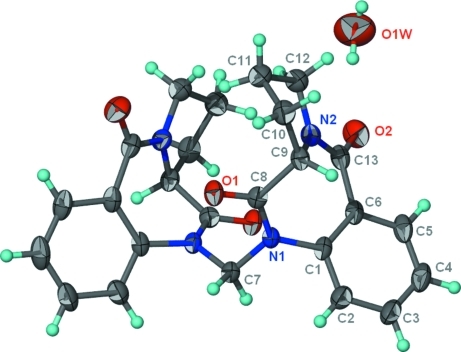
Thermal ellipsoid plot (Barbour, 2001) of C25H24N4O4.H2O at the 70% probability level; hydrogen atoms are drawn as spheres of arbitrary radius. Unlabelled atoms are related by a twofold axis.

## supplementary crystallographic information

### Experimental

2,3-Dihydro-1*H*-benzo[*e*]pyrrolo[1,2-*a*][1,4]diazepine-5,11(10*H*,11*aH*)-dione (1.1 g, 6 mmol), dibromomethane (1.8 ml, 2.54 mmol), potassium carbonate (0.71 g, 6 mmol) and a catalytic quantity of tetra-*n*-butylammonium bromide were stirred in *N*,*N*-dimethylformamide (20 ml) for 24 h. The insoluble salts were filtered off and the solvent was removed under vacuum. The residue was separated by chromatography on silica gel with an *n*-hexane:ethyl acetate (8:2) solvent system. The compound was obtained as colorless crystals in 70% yield upon evaporation of the solvent.

### Refinement

Carbon-bound H-atoms were placed in calculated positions (C—H 0.95 to 0.99 Å) and were included in the refinement in the riding model approximation, with *U*_iso_(H) set to 1.2*U*_eq_(C). The location of the water-bound H-atoms were ambiguous owing to disorder. The H1w1 atom was placed in a chemically sensible position on the basis of its hydrogen bonding to the O2 atom but was not refined. The water molecules are arranged along the c-axis with O1w···O1w separations of 2.741 (5) and 2.757 (5) Å, indicative of hydrogen bonding interactions. The assigned position for the second hydrogen atom (O-H = 0.84 Å), with full occupancy represents an intermediate position.

### Crystal data

**Table:**

C_25_H_24_N_4_O_4_·2H_2_O	*D*_x_ = 1.413 Mg m^−^^3^
*M**_r_* = 480.51	Mo *K*α radiation, λ = 0.71073 Å
Trigonal, *P*3_1_21	Cell parameters from 9284 reflections
Hall symbol: P 31 2"	θ = 5.0–28.3°
*a* = 11.9901 (2) Å	µ = 0.10 mm^−^^1^
*c* = 13.6054 (2) Å	*T* = 193 K
*V* = 1693.90 (4) Å^3^	Block, colorless
*Z* = 3	0.20 × 0.20 × 0.10 mm
*F*(000) = 762	

### Data collection

**Table:**

Bruker APEXII diffractometer	1472 reflections with *I* > 2σ(*I*)
Radiation source: fine-focus sealed tube	*R*_int_ = 0.043
graphite	θ_max_ = 28.3°, θ_min_ = 5.2°
φ and ω scans	*h* = −13→0
23041 measured reflections	*k* = 0→15
1572 independent reflections	*l* = 0→18

### Refinement

**Table:**

Refinement on *F*^2^	Secondary atom site location: difference Fourier map
Least-squares matrix: full	Hydrogen site location: inferred from neighbouring sites
*R*[*F*^2^ > 2σ(*F*^2^)] = 0.043	H-atom parameters constrained
*wR*(*F*^2^) = 0.126	*w* = 1/[σ^2^(*F*_o_^2^) + (0.0716*P*)^2^ + 0.3837*P*] where *P* = (*F*_o_^2^ + 2*F*_c_^2^)/3
*S* = 1.18	(Δ/σ)_max_ = 0.001
1572 reflections	Δρ_max_ = 0.46 e Å^−^^3^
159 parameters	Δρ_min_ = −0.48 e Å^−^^3^
0 restraints	Absolute structure: nd
Primary atom site location: structure-invariant direct methods	

### Fractional atomic coordinates and isotropic or equivalent isotropic displacement parameters (Å^2^)

**Table:**

	*x*	*y*	*z*	*U*_iso_*/*U*_eq_	Occ. (<1)
O1	0.57170 (18)	0.14709 (17)	0.22350 (11)	0.0274 (4)	
O2	0.7198 (2)	0.0623 (2)	0.57106 (13)	0.0362 (5)	
O1W	0.9339 (4)	0.0162 (4)	0.5795 (2)	0.0819 (10)	
H1W1	0.8758	0.0349	0.5692	0.123*	
H1W2	1.0069	0.0798	0.5645	0.123*	
N1	0.47771 (19)	0.1028 (2)	0.37359 (13)	0.0219 (4)	
N2	0.7341 (2)	0.16144 (19)	0.42630 (14)	0.0235 (4)	
C1	0.4814 (2)	0.1364 (2)	0.47532 (15)	0.0233 (5)	
C2	0.3891 (3)	0.1666 (3)	0.50682 (18)	0.0303 (5)	
H2	0.3310	0.1689	0.4606	0.036*	
C3	0.3812 (3)	0.1934 (3)	0.60537 (19)	0.0361 (6)	
H3	0.3195	0.2160	0.6261	0.043*	
C4	0.4645 (3)	0.1867 (3)	0.67300 (18)	0.0382 (7)	
H4	0.4581	0.2019	0.7407	0.046*	
C5	0.5571 (3)	0.1577 (2)	0.64133 (16)	0.0303 (6)	
H5	0.6133	0.1529	0.6882	0.036*	
C6	0.5699 (2)	0.1355 (2)	0.54191 (16)	0.0243 (5)	
C7	0.3561 (3)	0.0000	0.3333	0.0239 (6)	
H7A	0.2900	−0.0353	0.3858	0.029*	0.50
H7B	0.3253	0.0353	0.2809	0.029*	0.50
C8	0.5813 (2)	0.1646 (2)	0.31219 (16)	0.0224 (5)	
C9	0.7077 (2)	0.2487 (2)	0.36580 (15)	0.0225 (5)	
H9	0.7020	0.3141	0.4078	0.027*	
C10	0.8238 (2)	0.3128 (3)	0.29721 (17)	0.0296 (5)	
H10A	0.8938	0.3930	0.3265	0.036*	
H10B	0.7997	0.3332	0.2329	0.036*	
C11	0.8645 (3)	0.2109 (3)	0.28641 (18)	0.0320 (6)	
H11A	0.9549	0.2499	0.2641	0.038*	
H11B	0.8080	0.1425	0.2397	0.038*	
C12	0.8482 (3)	0.1579 (3)	0.39173 (18)	0.0317 (6)	
H12A	0.8334	0.0690	0.3918	0.038*	
H12B	0.9245	0.2133	0.4327	0.038*	
C13	0.6801 (2)	0.1157 (2)	0.51428 (16)	0.0251 (5)	

### Atomic displacement parameters (Å^2^)

**Table:**

	*U*^11^	*U*^22^	*U*^33^	*U*^12^	*U*^13^	*U*^23^
O1	0.0330 (9)	0.0290 (9)	0.0170 (7)	0.0132 (8)	−0.0008 (6)	0.0000 (6)
O2	0.0427 (11)	0.0404 (11)	0.0284 (9)	0.0229 (9)	−0.0039 (8)	0.0068 (8)
O1W	0.082 (2)	0.126 (3)	0.0681 (18)	0.075 (2)	−0.0051 (16)	0.0110 (19)
N1	0.0235 (9)	0.0246 (9)	0.0173 (8)	0.0117 (8)	−0.0014 (7)	−0.0033 (7)
N2	0.0260 (10)	0.0253 (10)	0.0196 (8)	0.0131 (8)	−0.0018 (7)	0.0005 (7)
C1	0.0273 (11)	0.0201 (10)	0.0184 (9)	0.0088 (9)	0.0018 (9)	−0.0020 (8)
C2	0.0304 (13)	0.0304 (12)	0.0296 (12)	0.0148 (11)	0.0025 (9)	−0.0054 (10)
C3	0.0323 (13)	0.0361 (14)	0.0334 (13)	0.0122 (12)	0.0061 (11)	−0.0124 (11)
C4	0.0365 (15)	0.0363 (14)	0.0242 (11)	0.0050 (12)	0.0091 (11)	−0.0074 (10)
C5	0.0330 (13)	0.0259 (12)	0.0178 (10)	0.0042 (10)	0.0009 (9)	0.0005 (9)
C6	0.0267 (11)	0.0209 (10)	0.0181 (10)	0.0064 (9)	0.0014 (8)	−0.0009 (8)
C7	0.0248 (11)	0.0276 (16)	0.0203 (13)	0.0138 (8)	−0.0021 (6)	−0.0041 (12)
C8	0.0271 (11)	0.0212 (10)	0.0206 (10)	0.0134 (9)	−0.0005 (8)	0.0010 (8)
C9	0.0271 (11)	0.0212 (10)	0.0173 (9)	0.0106 (9)	−0.0013 (8)	0.0014 (8)
C10	0.0287 (12)	0.0278 (12)	0.0247 (10)	0.0084 (10)	0.0034 (9)	0.0017 (9)
C11	0.0294 (12)	0.0387 (14)	0.0277 (12)	0.0168 (11)	0.0022 (10)	−0.0014 (11)
C12	0.0299 (13)	0.0382 (14)	0.0287 (12)	0.0183 (11)	−0.0029 (10)	−0.0031 (10)
C13	0.0293 (12)	0.0227 (11)	0.0193 (9)	0.0099 (9)	−0.0037 (9)	0.0002 (8)

### Geometric parameters (Å, °)

**Table:**

O1—C8	1.220 (3)	C5—C6	1.402 (3)
O2—C13	1.240 (3)	C5—H5	0.9500
O1W—H1W1	0.8429	C6—C13	1.502 (4)
O1W—H1W2	0.8500	C7—N1^i^	1.466 (3)
N1—C8	1.367 (3)	C7—H7A	0.9900
N1—C1	1.436 (3)	C7—H7B	0.9900
N1—C7	1.466 (3)	C8—C9	1.522 (3)
N2—C13	1.341 (3)	C9—C10	1.526 (3)
N2—C12	1.466 (3)	C9—H9	1.0000
N2—C9	1.485 (3)	C10—C11	1.533 (4)
C1—C2	1.393 (3)	C10—H10A	0.9900
C1—C6	1.399 (3)	C10—H10B	0.9900
C2—C3	1.393 (3)	C11—C12	1.540 (3)
C2—H2	0.9500	C11—H11A	0.9900
C3—C4	1.389 (4)	C11—H11B	0.9900
C3—H3	0.9500	C12—H12A	0.9900
C4—C5	1.387 (4)	C12—H12B	0.9900
C4—H4	0.9500		
			
H1W1—O1W—H1W2	109.8	O1—C8—N1	121.9 (2)
C8—N1—C1	122.97 (19)	O1—C8—C9	124.4 (2)
C8—N1—C7	118.68 (17)	N1—C8—C9	113.59 (18)
C1—N1—C7	118.32 (17)	N2—C9—C8	106.93 (18)
C13—N2—C12	122.8 (2)	N2—C9—C10	103.41 (19)
C13—N2—C9	123.6 (2)	C8—C9—C10	113.32 (18)
C12—N2—C9	111.90 (18)	N2—C9—H9	111.0
C2—C1—C6	120.4 (2)	C8—C9—H9	111.0
C2—C1—N1	116.9 (2)	C10—C9—H9	111.0
C6—C1—N1	122.6 (2)	C9—C10—C11	103.3 (2)
C1—C2—C3	120.7 (2)	C9—C10—H10A	111.1
C1—C2—H2	119.6	C11—C10—H10A	111.1
C3—C2—H2	119.6	C9—C10—H10B	111.1
C4—C3—C2	119.4 (3)	C11—C10—H10B	111.1
C4—C3—H3	120.3	H10A—C10—H10B	109.1
C2—C3—H3	120.3	C10—C11—C12	102.4 (2)
C5—C4—C3	119.8 (2)	C10—C11—H11A	111.3
C5—C4—H4	120.1	C12—C11—H11A	111.3
C3—C4—H4	120.1	C10—C11—H11B	111.3
C4—C5—C6	121.6 (2)	C12—C11—H11B	111.3
C4—C5—H5	119.2	H11A—C11—H11B	109.2
C6—C5—H5	119.2	N2—C12—C11	102.4 (2)
C1—C6—C5	117.9 (2)	N2—C12—H12A	111.3
C1—C6—C13	124.7 (2)	C11—C12—H12A	111.3
C5—C6—C13	117.4 (2)	N2—C12—H12B	111.3
N1^i^—C7—N1	109.8 (3)	C11—C12—H12B	111.3
N1^i^—C7—H7A	109.7	H12A—C12—H12B	109.2
N1—C7—H7A	109.7	O2—C13—N2	122.4 (2)
N1^i^—C7—H7B	109.7	O2—C13—C6	121.4 (2)
N1—C7—H7B	109.7	N2—C13—C6	116.2 (2)
H7A—C7—H7B	108.2		
			
C8—N1—C1—C2	124.6 (2)	C12—N2—C9—C8	113.4 (2)
C7—N1—C1—C2	−53.5 (3)	C13—N2—C9—C10	158.9 (2)
C8—N1—C1—C6	−57.4 (3)	C12—N2—C9—C10	−6.5 (2)
C7—N1—C1—C6	124.5 (2)	O1—C8—C9—N2	−112.1 (2)
C6—C1—C2—C3	−1.6 (4)	N1—C8—C9—N2	64.2 (2)
N1—C1—C2—C3	176.4 (2)	O1—C8—C9—C10	1.1 (3)
C1—C2—C3—C4	−1.6 (4)	N1—C8—C9—C10	177.5 (2)
C2—C3—C4—C5	2.1 (4)	N2—C9—C10—C11	29.1 (2)
C3—C4—C5—C6	0.4 (4)	C8—C9—C10—C11	−86.3 (2)
C2—C1—C6—C5	4.0 (3)	C9—C10—C11—C12	−40.6 (2)
N1—C1—C6—C5	−173.9 (2)	C13—N2—C12—C11	175.9 (2)
C2—C1—C6—C13	−174.4 (2)	C9—N2—C12—C11	−18.6 (3)
N1—C1—C6—C13	7.7 (4)	C10—C11—C12—N2	35.9 (2)
C4—C5—C6—C1	−3.4 (4)	C12—N2—C13—O2	−3.0 (4)
C4—C5—C6—C13	175.1 (2)	C9—N2—C13—O2	−166.9 (2)
C8—N1—C7—N1^i^	59.77 (17)	C12—N2—C13—C6	175.5 (2)
C1—N1—C7—N1^i^	−122.0 (2)	C9—N2—C13—C6	11.7 (3)
C1—N1—C8—O1	−169.7 (2)	C1—C6—C13—O2	−148.6 (2)
C7—N1—C8—O1	8.4 (3)	C5—C6—C13—O2	33.0 (3)
C1—N1—C8—C9	13.9 (3)	C1—C6—C13—N2	32.9 (3)
C7—N1—C8—C9	−168.02 (19)	C5—C6—C13—N2	−145.5 (2)
C13—N2—C9—C8	−81.2 (3)		

Symmetry codes: (i) *x*−*y*, −*y*, −*z*+2/3.

### Hydrogen-bond geometry (Å, °)

**Table:**

*D*—H···*A*	*D*—H	H···*A*	*D*···*A*	*D*—H···*A*
O1w—H1w1···O2	0.84	2.06	2.886 (4)	168
